# Decoding the immunological triad of TLRs, B1 cells, and feto-maternal microchimerism in pre-eclampsia pathogenesis: a narrative review

**DOI:** 10.3389/fimmu.2026.1906541

**Published:** 2026-07-23

**Authors:** Maithreyi Chandan Nair, Kesar Chandel, Shreevarsha S., Sneha Premraj, Nithya U., Gayathri M.

**Affiliations:** School of Bio-sciences and Technology, Vellore Institute of Technology, Vellore, Tamil Nadu, India

**Keywords:** angiotensin receptor autoantibodies, B1 cells, maternal-foetal tolerance, microchimerism, pre-eclampsia, toll-like receptors

## Abstract

Pre-eclampsia (PE) is a pregnancy-specific hypertensive disorder with fatal consequences for maternal and foetal health, including placental abruption, foetal growth restriction, and foetal death. Its global prevalence is estimated at 2-8%. While many aspects of PE pathogenesis are well-characterised, those pertaining to its immunology are less well understood. This review examines three interconnected aspects of the maternal immune microenvironment—toll-like receptors (TLRs), B1 cells, and feto-maternal microchimerism—and their role in PE pathogenesis. While each has been studied independently, no prior review has examined these three components as an interacting system. TLRs function as endogenous danger-molecule sensors, with pathological activation of TLR7 and TLR9 facilitating overzealous pro-inflammatory signalling, endothelial injury, and defective spiral-artery remodelling. B1 cells differentiate into a pathogenic phenotype during pathological pregnancies, secreting agonistic autoantibodies against the angiotensin II type 1 receptor (AT1-AA), associated with vasoconstriction and hypertension. Feto-maternal microchimerism generally serves to induce maternal immune tolerance towards foetal antigens. In PE pregnancies, increased trafficking of foetal cells has been shown to release cell-free foetal DNA into the maternal circulation, resulting in excessive activation of TLR-mediated sterile inflammation. This review explores the evidence for how these three factors may interact to create a positive feedback loop.

## 1 Introduction

Pre-eclampsia (PE) is a hypertensive disorder of pregnancy that is diagnosed after 20 weeks of gestation and characterised by new-onset hypertension (systolic ≥140mmHg or diastolic ≥90mmHg) and proteinuria (> 0.3 g/24 hours), or by end-organ dysfunction during the absence of proteinuria (1). Pre-eclampsia sits within a broader classification of hypertensive disorders of pregnancy, which also includes chronic hypertension, gestational hypertension, and chronic hypertension with superimposed pre-eclampsia. This framework is used to standardise diagnosis and guide differing management approaches across the disorders (2, 3). PE is further sub-classified by gestational age at onset, such as early-onset PE (EOPE), diagnosed before 34 weeks and is linked to specific pathophysiological mechanisms due to inadequate placentation, whereas late-onset PE (LOPE), identified at or after 34 weeks, is mainly thought to stem from maternal cardiovascular risk elements and placental insufficiency. This difference goes beyond just timing, as EOPE and LOPE vary in their fundamental pathophysiology, clinical progression, and maternal and neonatal outcomes (4). Eclampsia is characterised by the occurrence of generalised tonic-clonic seizures in a woman with PE, and HELLP syndrome (haemolysis, elevated liver enzymes, low platelets) represents severe manifestations along the same disease spectrum rather than separate disorders, and both are associated with substantially elevated maternal morbidity and mortality (5). According to the World Health Organisation (WHO), the prevalence of pre-eclampsia in pregnant women was found to be 2-8% worldwide. A cross-sectional study was conducted where 31 out of 500 pregnant women were found to have pre-eclampsia, accounting for 6.2% of the study population in India (6). Globally, PE remains a leading cause of maternal and neonatal mortality, particularly in low and middle-income countries (7). PE is classically understood through a two-stage model– Stage 1: Impaired trophoblast invasion during early gestation leads to shallow spiral artery remodelling, producing a hypoxic, ischaemic placenta. Stage 2: The stressed placenta releases anti-angiogenic factors, damage-associated molecular patterns (DAMPs), and pro-inflammatory mediators into the maternal circulation, resulting in endothelial dysfunction, hypertension, and proteinuria that define the disorder (1, 8). Immune dysregulation is increasingly recognised as central to both stages of PE pathogenesis rather than being a secondary consequence of placental ischaemia. The maternal-foetal interface presents a unique immunological challenge that the maternal immune system must simultaneously accommodate a genetically foreign foetus and preserve its capacity to fight infection. Any disturbance to this equilibrium, whether through excessive innate immune activation, breakdown of humoral tolerance or abnormal trafficking of cells between foetal and maternal compartments, can set off or sustain the systemic inflammatory cascade central to PE development (8, 9).

TLRs are pattern recognition receptors (PRRs) that can detect extracellular and endosomal patterns from the pathogen, such as pathogen-associated molecular patterns (PAMPs) and damage-associated molecular patterns (DAMPs), initiating an immune defence mechanism (10). TLRs are important mediators in inflammatory pathways that contain the Toll/IL-1 receptor (TIR) intracellular domain, which interacts with adaptor proteins to initiate cascade signalling reactions. TLR signalling proceeds through two independent pathways. Most TLRs use the myeloid differentiation primary response 88 (MyD88) pathway, where MyD88 binds the receptor’s TIR domain to activate nuclear factor kappa-light-chain enhancer of activated B-cells (NF-κB) and pro-inflammatory cytokine production. TLR3 signals exclusively, and TLR4 signals under certain conditions, via the MyD88-independent (TRIF-dependent) pathway, where the Toll/interleukin-1 receptor (TIR) domain-containing adaptor protein-inducing interferon-β (TRIF), aided by the TRIF-related adaptor molecule (TRAM) for TLR4, drives IRF3 activation and type I interferon production (11, 12). Both converge on inflammatory gene transcription but differ in kinetics and downstream effectors. In pre-eclampsia, the heightened TLR expression (particularly TLR7 and TLR9) can result in placental dysfunction, anti-angiogenic imbalance via vascular endothelial growth factor A (VEGF-A) and soluble fms-like tyrosine kinase-1 (sFlt-1), and systemic inflammation (13–16). B1 cells are a distinct subset of the B-cell lineage characterised by self-renewal capacity, constitutive immunoglobulin secretion, and high IL-10 production that plays a protective immunoregulatory role in normal pregnancy. In PE, B-1a cells shift towards a pathogenic phenotype and produce agonistic auto-antibodies against the angiotensin II type 1 receptor (AT1-AA), which have been implicated in vasoconstriction and hypertension due to aldosterone secretion. Their polyreactive nature predisposes them to cross-react with foetal antigens introduced via microchimerism, making them a key cellular mediator linking immune dysregulation to PE pathogenesis (17). Feto-maternal microchimerism refers to the two-way transfer of cells between mother and foetus, which normally supports the maternal immune tolerance to foetal antigens through regulatory T-cell networks (18). In pre-eclampsia, placental dysfunction and impaired invasion lead to the increased shedding of foetal cells, cell-free foetal DNA (cffDNA) and trophoblast-derived exosomes into maternal circulation, which have been proposed to activate TLR9 and contribute to pro-inflammatory signalling (19, 20). The interplay between TLRs, B1 cells and feto-maternal microchimerism creates a framework for a better understanding of immune-mediated mechanisms in PE. PE pathogenesis is multifactorial, and immune contributors beyond this triad, including decidual Natural killer cells, dendritic cells, complement activation, neutrophils, monocytes, the maternal microbiome, and oxidative stress mechanisms independent of reactive oxygen species (ROS), are also implicated and are reviewed extensively elsewhere. Some studies have explored the relationship between B1 cells and TLRs and their role in immune regulation during pregnancy. While individual roles of B1 cells, TLRs and feto-maternal microchimerism in pregnancy have been studied, no review to date appears to have these three systems interact as a unified immunological circuit in PE. This review addresses this gap by examining the individual roles of TLRs, B1 cells and feto-maternal microchimerism in normal pregnancy and in PE, rather than a comprehensive survey of PE immunology as a whole, before critically analysing the immunological circuit through which they interact, with implications for early biomarker detection, risk stratification and novel therapeutic targets in PE management.

## 2 Methodology

This review is a narrative mechanism-focused synthesis of the literature on the immunological triad of TLRs, B1 cells, and feto-maternal microchimerism in the setting of PE pathogenesis. It emphasises key biological domains, including TLR-driven inflammatory signalling in the placenta, B1 cell dysregulation and AT1-AA-mediated vascular pathology, feto-maternal cell trafficking and immune tolerance breakdown, and the convergent immunological circuit linking these three systems across disease progression.

Relevant studies were identified through systematic searches of major biomedical databases, including PubMed, Scopus, and Web of Science. The search strategy combined terms describing PE and its clinical sub-classifications (e.g., EOPE and LOPE, HELLP syndrome, hypertensive disorders of pregnancy) with terms related to the three immunological axes under review, such as TLR4/7/9, B1 cells, B-1a cells, AT1-AA, feto-maternal microchimerism, and cffDNA. Relevant peer-reviewed articles, foundational immunology papers, and clinical or cohort studies relating to PE were retrieved from 2003 to 2026, with greater emphasis on papers published within the last five years. Older studies were included selectively when considered foundational or highly relevant to establishing mechanistic context. Both human and animal-model studies were retrieved, given that a substantial portion of the mechanistic evidence in this field derives from murine models. Reference lists of selected articles were also screened to identify additional relevant publications.

## 3 B1 cells

### 3.1 Characteristic

B-cells are a diverse and functionally flexible subset of immune cells derived from haematopoietic stem cells in the foetal liver and, postnatally, bone marrow. They are crucial to humoral and cellular immunity as they produce antigen-specific antibodies, present antigens, provide co-stimulatory signals, and secrete cytokines that activate T-cells (9). There are several known subsets of B-cells, mainly B1 and B2 subsets, which have different ontogeny, anatomical localisation, and functions. B2 cells develop continuously from bone marrow precursors postnatally, undergo antigen-driven somatic hypermutation and class switching, and mature into follicular or marginal-zone B-cells, which are capable of forming long-lived memory and plasma cells. B1 cells, on the other hand, are primarily derived from foetal liver precursors during foetal and perinatal life, and can be distinguished by their self-renewal capacity, high IL-10 production, and secretion of protective natural antibodies. CD5 expression further divides B1 cells into B-1a (CD5+) and B-1b (CD5-) subsets. This classification is well-established in mice, but human B1 cells lack a single definitive marker, and CD5, CD27, and other markers are used inconsistently across human studies (21). B-1a cells are the predominant subset found in the peritoneal and pleural cavities. They are thought to be the main producers of natural IgM. However, they are also thought to be responsible for most autoimmune pathology due to their relatively germline-like repertoire and low rate of somatic hypermutation, leading to increased polyreactivity and cross-reactivity with self-antigens. Their polyreactivity can be beneficial, but under conditions such as pathological activation, like in PE, B-1a cells may be susceptible to producing auto-antibodies (9, 21).

### 3.2 Normal pregnancy

During pregnancy, B1 cells increase in number and take on an immunoregulatory phenotype, which is important for both maternal-foetal tolerance and a healthy pregnancy (21). While IL-10 is a key feature of this regulatory phenotype, it is not exclusive to B1 cells as regulatory T-cells, decidual macrophages and other B-cell subsets also contribute to the maternal IL-10 pool. B-1a cells should be understood as one of several sources rather than the central regulator of this pathway. This shift is driven in part by pregnancy-specific hormones. Human chorionic gonadotropin (hCG) has been shown to stimulate B-cells to produce IL-10, while α-fetoprotein promotes apoptotic clearance of auto-reactive B-cells, together fine-tuning the B-cell response toward tolerance. B1 cell populations are proposed to grow as IL-10 levels rise and tumour necrosis factor-α (TNF-α) production falls, promoting tolerance through higher serum IgM and IgG3 levels, which is demonstrated *in vitro* in murine models rather than directly confirmed in human pregnancy (22). These polyreactive antibodies passively shield the mother and foetus from harmful reactions, neutralise pathogens, and lower the risk of autoimmunity. B1 cell-derived IL-10 reduces complications related to inflammation by suppressing TNF-α, controls trophoblast invasion, and promotes placental artery growth (8, 23). The maternal immunity must tolerate the semi-allogenic foetus while shielding itself from outside threats. Despite comprising only 1-2% of decidual lymphocytes, B-cells are becoming more important for pregnancy maintenance through antibody production and regulatory functions (9).

### 3.3 Dysregulation during pre-eclampsia

Pre-eclampsia and other pregnancy complications are associated with B1 cell dysregulation. In contrast to normal physiological pregnancies, pre-eclampsia stimulates the B-1a cells to produce AT1-AA, which act as agonists to trigger signalling pathways that cause blood vessel constriction, aldosterone secretion, renin-angiotensin system (RAS) activation, and increased blood pressure—mechanisms discussed in detail in Section 6 (8, 9). This regulatory to pathogenic phenotype switch, detailed mechanistically in Section 6.3, is driven by convergent TLR and microchimeric antigen signals in the decidual environment (9). Several pieces of evidence support B-cell dysfunction in PE. Research on the peripheral B-cell populations in PE patients shows that there are increased frequencies of memory B-cells and plasma cell precursors as compared to healthy controls, along with a higher capacity for producing antibodies after they have been activated (24). More recently, a study has revealed that programmed cell death protein-1 (PD-1) expression has undergone substantial alteration in all of the naïve, transitional and memory B-cell subsets of women with PE, which also indicates that women with this condition have a dysregulation of the checkpoint control associated with humoral immunity (25). Furthermore, CD19+CD5+ B-cells have been identified specifically in the placental tissue of PE patients, where they correlate with local AT1-AA production (26). This finding, however, comes from a 2012 study; more recent work has since demonstrated that placental cells from women with PE can causally induce hypertension when adoptively transferred, which provides direct confirmation (27). Reported AT1-AA detection rates vary considerably across studies, from approximately 58% to 95% of PE cases, largely reflecting differences in assay methodology, where cardiomyocyte bioassays, luciferase reporter bioassays and ELISA are not directly comparable (28, 29).

The direction of B-cell dysregulation in PE is a topic of continued debate. Currently, much of the available information is correlated as an elevated AT1-AA and an enlarged population of B-1a is seen in both PE patients. The question of whether these changes in B-cells occur before the development of PE or as a result of inflammation due to placental ischaemia remains unanswered. Studies from animal models have shown that giving normal pregnant rats antibodies against AT1-AA can cause them to develop many of the characteristics of PE, suggesting a strong correlation between the presence of AT1-AA antibodies and the development of PE, implying a potential mechanism for the disease (8). To show this link in humans, prospective longitudinal studies using pre-symptomatic B-cell phenotyping will need to be conducted, which is currently a large gap in the literature.

## 4 Toll-like receptors

### 4.1 Characteristic

TLRs are type 1 transmembrane glycoproteins with extracellular leucine-rich repeat (LRR) motifs that mediate ligand recognition, and a cytoplasmic Toll/IL-1 receptor (TIR) domain that initiates intracellular signalling (10). Signalling proceeds through two distinct routes. Most TLRs act via myeloid differentiation primary response 88 (MyD88)-dependent pathway, while TLR3 and TLR4 can additionally signal through the independent TIR-domain-containing adaptor protein-inducing interferon-β (TRIF) and TRIF-related adaptor molecule (TRAM) pathway. Both converge on NF-κB and interferon regulatory factor activation (10, 12). Based on their cellular localisation and ligand specificity, TLRs are classified into two groups. Cell surface TLRs (TLR1, TLR2, TLR4, TLR5, and TLR10) mainly recognise microbial membrane components such as lipoproteins, lipopolysaccharides (LPS), and flagellin. Endosomal TLRs (TLR3, TLR7, TLR8, and TLR9), on the other hand, are present in the endoplasmic reticulum, endosomes, and lysosomes, and can recognise nucleic acids: TLR3 detects viral double-stranded RNA (dsRNA), TLR7 and TLR8 detect single-stranded RNA (ssRNA), and TLR9 detects unmethylated CpG motifs in bacterial DNA; cffDNA is also CpG-rich but carries a distinct methylation signature from bacterial DNA, and its recognition by TLR9 is not fully equivalent to that of microbial ligands (12, 30). TLRs respond to both microbial PAMPs and endogenous DAMPs, such as high mobility group box 1 (HMGB1) and heat shock protein 70 (HSP70), that are released during cellular stress. The ability of TLRs to recognise DAMPs during pregnancy is especially important, as placental stress produces a large quantity of DAMP signals and thus TLR-mediated sterile inflammation may occur even in the absence of infection (31, 32).

### 4.2 Normal pregnancy

Innate immune responses at the maternal-foetal interface can significantly influence the outcome of the pregnancy. This occurs because intrauterine infections are strongly associated with many pregnancy-related disorders (33). At this interface, all ten TLRs, along with co-receptors such as CD14, are expressed by immune cells, trophoblasts, decidual cells, and amniotic epithelium. Their expression is regulated in a spatially and temporally dynamic manner during the gestation period: TLR expression is relatively restrained in the first trimester to allow implantation and trophoblast invasion, increases through the second trimester for antimicrobial protective services associated with placental expansion, and is attenuated near term to prevent unwanted inflammatory activation before physiological labour (31, 34).

Cell-surface and intracellular TLRs lead to intra-amniotic inflammatory responses, antiviral defence via type I interferon production, dendritic cell maturation, and regulation of key immunologic events, like implantation and the onset of labour (31). Under specific physiological conditions, especially exposure to low physiological levels of LPS that may arise from normal gut microbiome translocation, TLR4 promotes a balanced cytokine environment that supports trophoblast differentiation and maternal-foetal tolerance (35, 36).

### 4.3 Dysregulation during pre-eclampsia

During pre-eclampsia, TLR expression and signalling are altered in a subtype- and stage-specific manner. Recent studies show that all TLRs remain present in PE but exhibit differential changes depending on the disease onset. In EOPE, surface TLR1 and TLR4 are paradoxically downregulated in placental tissue, while endosomal TLR7, TLR8, and TLR9 are significantly upregulated. LOPE shows comparatively subtler TLR expression changes, suggesting different underlying immunopathological triggers, which is an important distinction for biomarker development and therapeutic targeting (1, 25). This apparent paradox of TLR4 downregulation in EOPE alongside its role in pathological inflammation still needs to be examined. It should be noted that the findings on TLR4 expression in PE are inconsistent across the literature. Some studies report downregulation as described here, while others report TLR4 upregulation in placental or peripheral tissue. This discrepancy likely reflects differences in gestational timing, tissue compartment sampled (placental vs peripheral blood) and PE subtype (EOPE vs LOPE), rather than a single consistent expression pattern. Under pathological conditions, such as increased concentrations of LPS arising from subclinical infection or gut microbiota imbalances, TLR4 ligation inhibits trophoblast migration and impairs the remodelling of the spiral arteries. In addition, TLR4 ligation also leads to the activation of the NF-κB-driven cytokine cascades, which promote oxidative stress and endothelial dysfunction (35, 36). The surface downregulation is likely due to either internalisation of the TLR4 receptor or due to the negative regulatory feedback following a previous chronic TLR4 stimulation, rather than a reduction in TLR4-mediated inflammatory output (35). Among endosomal TLRs, TLR9 activation by cffDNA is associated with suppression of VEGF-A and upregulation of sFlt-1, mediated in part through HIF-1α stabilisation and downstream cytokine signalling, ultimately reducing angiogenesis and spiral-artery remodelling (14). TLR7 overactivation in EOPE placental tissue correlates with impaired trophoblast migration and has been reported to activate pro-apoptotic Phosphoinositide 3-kinase (PI3K) - Protein kinase B (PKB/Akt) and p53 pathways, based on evidence synthesised in a review by Banerjee et al. (32).

It should be noted, however, that most of the mechanistic studies conducted with TLRs are performed in murine models and *in vitro* trophoblasts, and currently, only a few human-based studies confirming the causal relationships have been conducted. Additionally, stage-stratified clinical trials must be done before TLRs can be utilised as therapeutic targets.

## 5 Microchimeric cells

### 5.1 Characteristic

Microchimerism is the presence of small and genetically distinct cells from a different individual within the host, which commonly arises during pregnancy when an exchange of cells occurs between the mother and the foetus. This can persist for decades and has been linked to physiological and pathological roles, including those in autoimmune disease, cancer, and wound healing (37, 38). There are two types of microchimerism: natural and artificial. Natural microchimerism includes foetal microchimerism (FMC), maternal microchimerism (MMC), and grandmaternal microchimerism. In FMC, intact foetal haematopoietic, immune, and stem cells enter and persist in the maternal circulation. In MMC, maternal cells cross the placenta into foetal tissues and may persist postnatally (39, 40). Grandmaternal microchimerism refers to the DNA and cells from the maternal grandmother detected at very low levels in grandchildren. Although this suggests a third-generation cell transfer, this still has uncertain immunological and clinical implications. Emerging evidence suggests that it may carry intergenerational effects on immune regulation, and its association with PE is an active area of investigation (18, 41). Artificial microchimerism occurs via external sources, such as organ transplantation, blood transfusions, or stem cell therapies, where the donor cells persist in the recipient. This is less common than pregnancy-derived microchimerism (38, 42). Human microchimerism studies vary considerably in detection method (qPCR, FISH, flow cytometry) and sample size, limiting direct comparability of prevalence estimates across the literature.

### 5.2 Normal pregnancy

Microchimerism, especially FMC, helps in inducing maternal immune tolerance to the semi-allogenic foetus by maintaining the immune regulatory networks, including regulatory T-cells. This reduces the risk of maternal immune attack against the foetus (40, 42). Similarly, the MMC builds the foetal immune system by promoting tolerance to maternal antigens. It also has a role in neurodevelopment and in the search for biomarkers of neurodevelopmental disorders (43, 44). Recent works on grandmaternal microchimerism suggest possible generational immune effects, although this is still an emerging field (41). FMC in maternal tissues can behave as stem-like cells and are home to sites of injury, where they integrate into maternal organs and support tissue repair and recovery from pregnancy- or birth-related damage. Fatal mesenchymal stem-like microchimeric cells interact with trophoblasts to promote placental development and proper implantation (45, 46). Persistence of these cells after pregnancy may influence future pregnancies and long-term maternal immune function and tissue health (37). Whether this persistence is primarily beneficial, neutral, or potentially harmful depends on the immunological context, something that is central to understanding microchimerism’s role in PE.

### 5.3 Dysregulation during pre-eclampsia

Many studies show that FMC levels are higher in women with PE as compared to healthy pregnancies, with Gammill et al. (2013) reporting that 32% of PE patients had detectably elevated foetal microchimerism compared to healthy pregnant controls (47). More recent work has confirmed this and further established that elevated FMC is particularly pronounced in early-onset PE and correlates with disease severity (18, 20). There are two possible pathways for pathological effects from elevated concentrations of FMC. The first is foetal cell death and damage to the placenta, which causes cffDNA, containing an abundance of unmethylated CpG, to be released into maternal circulation. The presence of cffDNA in the circulation activates TLR9, resulting in the activation of MyD88-NF-κB signalling and up-regulation of pro-inflammatory cytokines along with sFlt-1. This cffDNA-TLR9 axis is discussed in detail in Section 6 (14, 19). Secondly, the composition of microchimeric cells in PE shifts towards foetal immune subsets (such as CD4+ T cells and natural killer (NK) cell precursors) as opposed to having both stem-like and mesenchymal cell populations, which are more prevalent in healthy pregnancies. This is associated with greater maternal immune activation in PE compared with healthy pregnancies (8, 20).

Dysregulated microchimerism may compromise maternal immune tolerance by depleting decidual Treg populations that would otherwise suppress anti-foetal cytotoxic responses, which would subsequently contribute to the Th17-based pro-inflammatory state associated with PE, and is another potential mechanism by which dysregulation could affect the placental immune environment (8, 18). Whether elevated FMC is a primary driver of PE or arises secondarily from placental dysfunction remains an open question and an area of focus for future research.

## 6 Immunological interplay

### 6.1 The initiation axis: foetal microchimerism, cffDNA and TLR activation

In early PE, placental dysfunction and hypoxia impair the integrity of the placental barrier. This leads to increased trafficking of foetal cells into maternal circulation while simultaneously elevating cffDNA (39, 40). The cffDNA is enriched in unmethylated CpG motifs compared to maternal DNA, though the methylation pattern, which is a characteristic feature of foetal DNA methylation patterns, varies, and this distinction is not absolute, such that the degree of unmethylation influences the extent of TLR9 recognition (19, 48). The CpG-rich fragments are potent endogenous agonists and recognise TLR9, which is expressed on trophoblasts, decidual macrophages, NK cells and dendritic cells at the maternal-foetal interface. TLR9 engagement leads to the activation of MyD88-NF-κB signalling and upregulation of pro-inflammatory cytokines (14, 19, 49). cffDNA-driven TLR9 signalling induces the production and secretion of TNF-α, IL-6 and IL-8 in trophoblasts. This inflammatory cascade process includes downstream mediators such as HIF-1α, which has also been linked to upregulation of sFlt-1, which sequesters VEGF-A and disrupts angiogenesis (19). Higher total cffDNA levels correlate with earlier onset and more severe pre-eclampsia, which may point to a significant role for cffDNA-driven inflammation in disease progression (32, 50). Elevated foetal cell trafficking releases DAMPs, such as HSP70 and HMGB1, which, alongside excess cffDNA and foetal cell debris, function as an alarmin to activate PRRs such as TLR2 and TLR4 on trophoblasts, dendritic cells and macrophages, thus amplifying sterile inflammation in pre-eclampsia (32, 51).

### 6.2 The TLR-driven immune cascade: macrophage polarisation, Treg/Th17 imbalance and cytokine storm

Hofbauer cells (placental macrophages) of the placental stroma play a crucial role in promoting maternal tolerance towards the foetus by modulating the immune response. In a normal pregnancy, the macrophage M2 phenotype supports immune tolerance and trophoblast function. In pre-eclampsia, activation of TLR4 and endosomal TLRs by DAMPs at the placental interface has been associated with a shift in macrophage polarisation from M2 to M1, resulting in the production of pro-inflammatory mediators including TNF-α and IL-6, ROS and additional DAMPs that enhance the local inflammatory cascade and contribute to cytokine storm (52). TLR activation, particularly via TLR2 and TLR4, has also been linked to disruption of the regulatory T-cells (Treg)/T-helper 17 cells (Th17) balance that is essential for maternal-foetal tolerance. This is proposed to occur via NF-κB-mediated production of IL-6 and IL-23, which may increase Th17 differentiation while impairing Forkhead box protein (FoxP3)-dependent Treg induction. This alteration of the Treg/Th17 ratio, which favours Tregs during healthy pregnancy towards a Th17-dominant state, has been proposed to enhance systemic inflammation through IL-17-mediated endothelial activation, neutrophil recruitment and sustained pro-inflammatory cytokine release, contributing to the immune dysregulation associated with PE pathogenesis. It is important to recognise that Th17 responses are not consistently pathological in pregnancy. The protective Th17 subsets have also been described, and the functional balance between pathogenic and protective subsets in PE remains incompletely characterised. Overactivation of TLR7 and TLR9 by cffDNA and viral nucleic acid has been shown, in murine models, to suppress Treg differentiation, while the co-presence of IL-6 and transforming growth factor-β (TGF-β) induced by TLR7 and TLR9 overactivation is proposed to redirect naive T-cell differentiation away from the Treg lineage towards Th17 expansion (8, 52). Th17 cells produce IL-17, which has been linked to endothelial inflammation, vascular hyperpermeability and oxidative stress, features associated with the maternal systemic syndrome in PE. This proposed shift from Treg to Th17 might diminish the immunological restraint on B-cell activation, potentially contributing to conditions favourable for B-1a cell-driven autoantibody production (9). It should be noted that Treg/Th17 imbalance is not the sole T-cell abnormality implicated in PE, other subsets such as γδ T cells and Natural killer T cells have also been reported, though their relationship to the TLRmicrochimerism circuit discussed here remains largely unexplored.

### 6.3 The B1 cell activation loop: AT1-AA production and vascular pathology

B-1a cells in the decidua and peripheral blood are activated through two convergent signals in the context of elevated microchimerism and TLR dysregulation. First, TLR3 and TLR7 stimulation has been shown, in murine models, to activate B-1a cells, shifting them from a regulatory (high phosphatidylcholine 1 (PC1)/IL-10) to a pathogenic (low PC1/IL-10) phenotype, demonstrated by Schumacher et al. (2018), who identified two functionally opposed B-1a subsets in murine models defined by PC1 expression (53). Second, feto-maternal microchimeric antigens that include paternal human leukocyte antigen (HLA) molecules on foetal immune cells that have trafficked into the maternal decidua provide antigenic stimulation that, in the absence of Treg suppression, has been proposed to drive B-1a cell autoantibody production (9, 20).

The specific surface markers defining human B-1a cells remain poorly characterised. Unlike in murine models, where CD5 reliably distinguishes B-1a cells from the B-1b population, no consensus marker set has been established for human B1 cells. CD19+CD5+ has nonetheless been used provisionally in some preliminary studies, including reports of this population in pre-eclamptic placentas. These cells are reported to be capable of producing AT1-AAs. The resulting AT1-AA has been proposed to bind agonistically to the AT1 receptor on vascular smooth muscle cells, activating the RAS, including aldosterone secretion, and has been linked to vasoconstriction and stimulation of NF-κB as a downstream target, with associated transcription of pro-inflammatory mediators, including IL-6 and TNF-α (25, 26). The AT1-AA has also been linked to endothelin-1 production and nicotinamide adenine dinucleotide phosphate (NADPH) oxidase activation, generating ROS that may damage the endothelium and impair nitric oxide-mediated vasodilation, which has been associated with the hypertension and endothelial dysfunction of PE (8). Reported detection rates of AT1-AA in PE vary considerably across studies, ranging from approximately 70% to 95%, likely reflecting differences in assay methodology, patient population, and disease severity at sampling (28, 54). This variability has not been fully resolved and limits the current interpretation of AT1-AA as a standalone marker. In pre-eclampsia, elevated AT1-AA correlates with disease severity and has been proposed as a biomarker of EOPE. However, independent diagnostic utility remains limited and further validation in larger clinical samples remains necessary (55).

### 6.4 The positive feedback loop: ROS, DAMPs and continued TLR activation

**Figure 1:**
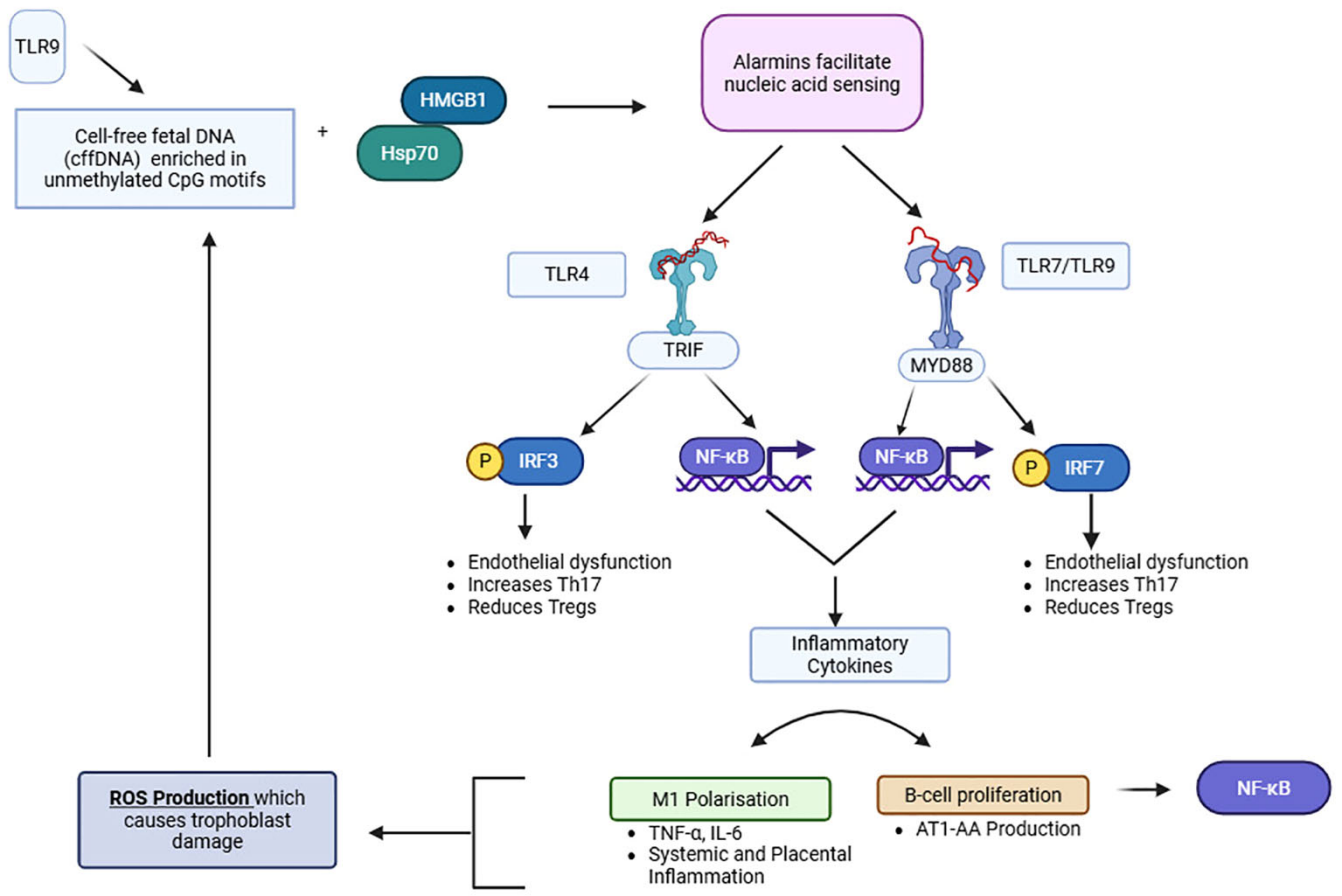
Schematic of TLR4/TLR7/TLR9 signalling triggered by cffDNA and alarmins in pre-eclampsia. TLR4 senses HMGB1/HSP70. TRIF signals via IRF3, while MyD88 signals via NF-κB and IRF7, both linked to endothelial dysfunction and Th17/Treg imbalance. Downstream cytokines drive M1 macrophage polarisation and B-1a cell expansion, the latter linked to AT1-AA output. Resulting ROS injures the trophoblast, feeding further DAMPs/cffDNA back into the same receptors.

Critically, the vascular damage inflicted by AT1-AA and ROS is not self-limiting. ROS-induced endothelial and trophoblast damage generates further DAMP release (HMGB1, HSP70, oxidised phospholipids), which has been proposed to re-engage TLRs, potentially sustaining the inflammatory signal (32, 51). Simultaneously, the systemic inflammation produced by this cascade may further impair placental function, increasing foetal cell death and cffDNA release, which has been proposed to reactivate TLR9, feasibly completing this positive feedback loop (19). Elevated ROS has also been linked to B-1a cells activation and survival, presumably maintaining AT1-AA production (9). The described self-amplifying circuit, in which microchimerism-driven TLR activation, macrophage polarisation, Treg/Th17 imbalance, and B-1a-mediated AT1-AA production are proposed to reinforce one another, represents a hypothesised model rather than an established mechanism, and may contribute to progressive immunological and vascular deterioration in PE. This proposed circuit describes a loop, beginning with cffDNA and DAMP-driven TLR4/TLR7/TLR9 activation, proceeding through TRIF-dependent and MyD88-dependent macrophage polarisation and Treg/Th17 imbalance, and ROS-driven endothelial damage that regenerates the initiating signal (**Figure 1**). This integrated model illustrates how feto-maternal microchimerism, alarmin release, and TLR signalling may converge to sustain the chronic inflammatory and vascular phenotype characteristic of PE, though as discussed below, it remains a hypothesised framework requiring further validation.

### 6.5 Translational limitations

A key limitation of the proposed circuit is that most mechanistic evidence comes from animal models and *in vitro* systems. This raises questions about the specific contribution of each pathway in human PE, including the order in which they occur, which is not clearly defined. The link between TLR signalling and B-1a cells phenotype switching has been established mainly in murine peritoneal B-1a cells, and whether this holds true in the human decidua and peripheral B-cell compartment remains an open question, warranting further investigation (9, 53). This uncertainty is compounded by known interspecies divergence in B1/B-1a cell biology. Human B-1-like cells differ from their murine counterparts in surface marker expression, tissue distribution, and repertoire features such as somatic hypermutation, and their equivalence to murine B-1a cells remains actively debated (56). A further complication is the clinical heterogeneity of PE itself. EOPE and LOPE are increasingly recognised as mechanistically distinct entities with differing placental, angiogenic, and immunological profiles (57, 58). In an observational study, Broekhuizen et al. found that placental TLR1 and TLR4 expression was reduced in early-onset but not late-onset, indicating that the innate-immune arm of the proposed circuit may not apply uniformly across PE subtypes (57). It remains unclear whether the murine-derived circuit accurately reflects any of these clinical phenotypes. Resolving these gaps will likely require paired human sampling strategies, such as matched decidual and peripheral blood collection across gestation in prospective cohorts, combined with single-cell transcriptomic profiling of B-cell subsets, an approach increasingly used to characterise the maternal-foetal interface in other pregnancy implications (59).

## 7 Discussion

### 7.1 Early detection and risk stratification

Early identification of women at high risk for pre-eclampsia is important for implementing preventive measures. Risk factors like a history of pre-eclampsia, chronic hypertension, renal diseases, autoimmune disorders, and certain metabolic conditions are checked for during the early stages. Screening early during pregnancy allows timely interventions, like prescribing low-dose aspirin, which has been shown in various studies to help reduce the incidence of pre-eclampsia among high-risk pregnancies (60). Other recent advancements include the introduction of novel pharmacological candidates like statins and metformin, which have been shown to reduce the risk. These agents need further trial evidence before routine use. Integrating validated biomarkers and risk calculators can enhance the accuracy of risk stratification, thus enabling targeted prevention strategies (61).

### 7.2 Diagnostic and predictive biomarkers

#### 7.2.1 TLR-based markers

Protein studies in placental tissues from individuals with EOPE showed an increased expression of TLR7, which has been linked to compromised trophoblast migration and proliferation—both essential for normal placental development—by the activation of downstream pro-apoptotic pathways such as PI3K-Akt and p53, which cause placental ischaemia and aberrant vascular remodelling (32). Elevated blood TLR4 and its associated NF-κB p65 can act as biomarkers that differentiate pre-eclampsia from healthy pregnancies, while increased TLR9-driven responses act as a non-invasive marker of disease presence and severity (35). Beyond diagnostic differentiation, Elevated TLR7 and TLR4 levels are furthermore associated with adverse outcomes, including preterm delivery, severe hypertension, and foetal growth restriction (32). However, their current clinical usage is decreased by technical assay variability and differences between EOPE and LOPE (1). Before incorporating TLR-based markers into clinical practice, more extensive, standardised validation studies are needed.

#### 7.2.2 B1 cell population as a biomarker

Several studies suggest that the distribution of B-cell subsets differs greatly between pre-eclampsia patients and healthy controls, with pre-eclamptic women exhibiting an increased concentration of B-1a cells involved in disease-associated immune activation (55). Altered activation status and changes in PD-1 expression across naïve, transitional, and memory B-cell subsets also show dysregulated humoral immunity in pre-eclampsia (25). These B-cell alterations have been connected with clinical severity markers, like elevated blood pressure and proteinuria, showing their potential use in risk assessment (14, 62). However, B-1a cell frequencies don’t always distinguish between mild and severe disease, limiting their independent diagnostic value. The clinical relevance of B-1a cells extends beyond immune profiling, as they are the primary producers of AT1-AA (Section 6.3). AT1-AA’s reported detectability in maternal serum has been proposed as a feasible non-invasive biomarker, although its diagnostic sensitivity is inconsistent across the different PE subtypes (26, 55). Similar to TLR-based markers, AT1-AA measurement and B-1a cell profiling are best seen as parts of a multimarker panel rather than as independent diagnostic tests.

#### 7.2.3 Microchimerism patterns and genetic ancestry

Building on the heightened foetal microchimerism (FMC) levels in PE discussed in Section 5.3, levels of FMC vary across populations and are influenced more strongly by genetic ancestry than by ethnicity (45, 46). The connection between genetic ancestry and the risk of microchimerism-related PE is a developing field. Variations in immune-regulatory gene polymorphisms, placental function, and HLA diversity among ancestral backgrounds might be the reason for some of the observed variation in PE incidence and severity between populations (63, 64). Ancestry-informed immunological profiling is still a promising but insufficiently established method for PE risk models. Prior to the diagnostic application of any ancestry-based microchimerism marker, population-specific validation studies are required.

### 7.3 Therapeutic targeting opportunities

#### 7.3.1 Targeting angiogenic imbalance

Expanding upon the sFlt-1–VEGF/PlGF imbalance discussed in Section 6.1, one therapeutic approach is to increase circulating PlGF levels to competitively offset sFlt-1 sequestration. The therapeutic potential of this strategy was supported by Spradley et al. (2016), who showed that exogenous PlGF treatment eliminated ischaemia-induced hypertension in a rat model of placental ischaemia, but this evidence is only from a rat model, and clinical translation awaits human pregnancy safety data (65). When paired with sFlt-1 ratios, PlGF also may contribute to usefulness as a diagnostic biomarker, but this evidence requires more clinical validation (66). Another experimental strategy involves removing circulating sFlt-1 using apheresis columns; in a pilot randomised controlled trial, Thadhani et al. (2016) showed that extracorporeal sFlt-1 removal somewhat extended pregnancy and improved clinical status in women with preterm PE, but the strategy has not been routinely used in clinical settings since it entails maternal procedural risk and lacks large-scale trial evidence (1). Gene therapy techniques that target sFlt-1 expression in the placenta constitute a longer-term strategy but still lie in the early preclinical stages with no established pregnancy safety data or regulatory pathways (67). Although none of these approaches is currently intended to take the place of existing PE management—including low-dose aspirin, antihypertensive therapy, magnesium sulphate, and timely delivery—they could potentially be used as supplementary strategies once validated.

#### 7.3.2 Targeting the B1 cell -AT1-AA axis

As it is strongly linked to the vasoconstriction and hypertension of PE and is situated downstream of several converging immunological signals, the AT1-AA pathway (produced by dysfunctional B-1a cells, as previously discussed in section 6.3) represents a therapeutically appealing target. It is demonstrated that B-cell depletion utilising anti-CD20 (rituximab-equivalent) techniques lowers AT1-AA levels and attenuates PE-associated hypertension in animal models, offering proof-of-concept for B-cell-targeted therapy. However, broad B-cell depletion limits its therapeutic relevance during pregnancy. This strategy involves a risk of infection for the mother and unclear foetal safety (8). In experimental systems, direct AT1-AA neutralisation with peptide-based competitive inhibitors has been examined in preclinical/*in vitro* systems only, with no human pregnancy safety data available (8, 9). A more mechanistically thorough strategy is to target the upstream B-1a cell activation signals, such as TLR3/7 signalling and the Treg/Th17 imbalance, which may also indirectly lower AT1-AA synthesis. Mesenchymal stem cells (MSCs) and regulatory B-cell infusion are examples of immunomodulatory cell-based therapies that could be used to improve immunological tolerance at the mother-foetal interface, but these methods are still in the preclinical phase with unresolved concerns regarding foetal safety and immunogenicity (8). Similar to angiogenic-targeted approaches, none of these approaches is currently intended to take the place of existing PE management—including low-dose aspirin, antihypertensive therapy, magnesium sulphate, and timely delivery—but they could potentially be used as supplementary strategies once validated.

### 7.4 Future directions

Our understanding of the immune microenvironment in pre-eclampsia is ever-evolving, and with time, it keeps growing in terms of research as well as technical advances. Several key research gaps emerge from the framework developed in this review, each with direct implications for understanding the TLR-B1 cell-microchimerism triad in PE (8, 37).

- Single-cell and spatial profiling: Single-cell technologies, like CyTOF mass cytometry and scRNA-seq, should be applied to placental and decidual tissue from EOPE and LOPE patients to characterise the specific microchimeric cell subtypes, macrophage polarisation states, and B-cell activation stages that distinguish disease subtypes, resolving a key limitation of current bulk tissue studies (8, 68).
- Immune crosstalk: There is currently a significant lack of prospective longitudinal studies that profile TLR expression, B-1a cell phenotype, AT1-AA levels, and quantitative FMC during normal pregnancy simultaneously. Such studies are necessary to determine the temporal sequence of the immune events before the onset of PE and whether dysregulated microchimerism, TLR activation, or B-1a cell switching is the initiating event (9, 20, 31, 68).
- Tissue-based immune profiling: Apart from maternal peripheral blood analysis, examining the immune composition of the placental bed throughout gestation is needed, as it will give us a better understanding of PE pathogenesis. This is because maternal blood does not fully capture the local processes involved in the development of PE (25, 46, 68).
- Therapeutic targeting: Modulation of TLR4/TLR9 signalling and autoantibody pathways, along with cell-based approaches such as MSCs or immunomodulatory B-cells, remains under study. These cell-based approaches remain preclinical, with no safety data in pregnant populations to date, and any intervention must avoid disrupting the immune activation required for normal labour. More importantly, any immunomodulatory intervention must consider gestational timing because there needs to be an adequate amount of immune activation close to delivery for normal labour to occur (35, 36).
- Microchimerism as a biomarker: FMC quantification from maternal blood may provide a non-invasive measure that strongly correlates with the severity of PE, making it a good candidate for incorporation into first-trimester screening panels. No FMC assay is currently validated for clinical use, and its performance relative to the existing sFlt-1/PlGF standard, along with cost and feasibility of implementation, still remains to be established before screening-panel inclusion can be considered realistic. It warrants further investigation for its role as an immune modulator and mediator of long-term health outcomes for mothers and their babies (18, 20, 37).

### 7.5 Concluding remarks

Pre-eclampsia is a complex, multifactorial condition in which dysregulated immune activity at the maternal-foetal interface significantly contributes to the progression towards eclampsia and is the active driver of its pathogenesis. Through examining the existing literature, this review has elucidated how the three immunological components, TLRs, B1 cells and feto-maternal microchimerism together form an interconnected, self-reinforcing circuit that sustains sterile inflammation, impairs angiogenesis and promotes vascular injury in PE. Specifically, dysregulated FMC notably increases cffDNA release, which activates the Treg/Th17 balance, promotes M1 macrophage polarisation, and permissively activates B-1a cells. These AT1-AAs derived from B-1a cells induce vasoconstriction, RAS activation and ROS-mediated endothelial damage, whose products in turn generate additional DAMPs that re-engage TLRs and re-amplify the cycle. These NF-κB act as a common downstream transcription factor integrating signals from both TLRs and B-cell receptors.

Animal studies have provided supporting evidence for AT1-AA pathogenesis, along with B-cell depletion that reduces hypertension and PE-related symptoms in rodent models. However, the translational gap between experimental and human data remains significant, and the causal sequence of events in human PE has not been definitively determined. Resolving this through prospective long-term immunological studies will be essential for identifying the precise checkpoints in TLR signalling, B-cell activation or foetal cell trafficking, where targeted intervention could interrupt the pathological cycle before clinical PE develops. This framework directly affects precision medicine in obstetric care, as integrating TLR expression, AT1-AA levels and quantitative microchimerism data into comprehensive multimarker panels could significantly enhance early risk assessment. In contrast, mechanistically targeted immunomodulatory therapies that are directed at TLR4/TLR9 signalling, B-1a cell activation or angiogenic imbalance offer promising avenues for future clinical development.
